# Sex-Specific Differences in Chronic Thromboembolic Pulmonary Hypertension Treated with Balloon Pulmonary Angioplasty

**DOI:** 10.3390/jcm14030899

**Published:** 2025-01-29

**Authors:** Paweł Kurzyna, Anna Witowicz, Piotr Kędzierski, Michał Florczyk, Marta Banaszkiewicz, Piotr Szwed, Michał Piłka, Aleksandra Gąsecka, Arkadiusz Pietrasik, Adam Torbicki, Marcin Kurzyna, Szymon Darocha

**Affiliations:** 1 Department of Pulmonary Circulation, Thromboembolic Diseases and Cardiology, European Health Center Otwock, Centre of Postgraduate Medical Education, ERN-LUNG Member, 05-400 Otwock, Poland; 2 Doctoral School of Translational Medicine, Centre of Postgraduate Medical Education, 01-813 Warsaw, Poland; 3 Department of Cardiology and Internal Diseases, Military Institute of Medicine—National Research Institute, Szaserow Street 128, 04-141 Warsaw, Poland; anna.witowicz@gmail.com; 4 Department of Vascular, Endovascular Surgery, Angiology and Phlebology, Poznan University of Medical Science, 61-701 Poznań, Poland; 5 1st Chair and Department of Cardiology, Medical University of Warsaw, Banacha 1a, 02-097 Warsaw, Poland; 6 Amsterdam Vesicle Center, Amsterdam University Medical Center, University of Amsterdam, 1012 Amsterdam, The Netherlands

**Keywords:** pulmonary hypertension, chronic thromboembolic pulmonary hypertension, balloon pulmonary angioplasty, sex-specific differences

## Abstract

**Background/Objectives:** Several studies describe the sex-specific differences in cardiovascular diseases. However, there is still limited research reporting the difference between men and women with chronic thromboembolic pulmonary hypertension (CTEPH) treated with balloon pulmonary angioplasty (BPA). The aim of this study was to evaluate sex-specific differences in patients with CTEPH treated with BPA. **Methods:** This retrospective study included CTEPH patients treated with BPA. The patients’ hemodynamic and clinical parameters were assessed at baseline and 3 months after completion of BPA treatment. **Results:** This study included 94 patients (44 women, 46.8%). At baseline, women had higher systolic pulmonary arterial pressure (sPAP) (76 ± 18.5 vs. 85 ± 17.6 mmHg; *p* = 0.03) and pulmonary vascular resistance (8.21 [5.55–10.17] vs. 9.89 [6.31–14.06] Wood Units; *p* = 0.03) compared to men. There were no differences in clinical characteristics between the sexes. At follow-up, women had lower sPAP (49 [41–54] vs. 43 [37–49] mmHg; *p* = 0.04) and pulmonary capillary wedge pressure (10 [9–14] vs. 9 [8–11] mmHg; *p* = 0.03), but a higher cardiac index (2.57 ± 0.53 vs. 2.82 ± 0.50 L/min/m^2^; *p* = 0.03), as well as better Dyspnea Borg Scale outcomes, compared to men. Women had a greater reduction in mean pulmonary artery pressure (−43% vs. −37%; *p* = 0.049) than men. **Conclusions:** At baseline, women with CTEPH had worse hemodynamic parameters than men despite similar clinical symptoms. However, the hemodynamic status of women was better after BPA therapy. Hence, women seem better adapted to the disease at baseline and respond better to BPA. Further data are needed to investigate whether the management of CTEPH patients should be sex-differentiated.

## 1. Introduction

Cardiovascular diseases (CVDs) remain the leading cause of death among women and men worldwide [1]. In most clinical trials in cardiology, men dominate, and women are underrepresented. However, in the case of various forms of pulmonary hypertension (PH), the opposite is true: women predominate quantitatively among the participants in clinical trials [2,3,4,5]. This is due to the fact that women are more susceptible than men to several forms of PH [6,7].

Chronic thromboembolic pulmonary hypertension (CTEPH) is a condition defined by elevated pressure in the pulmonary vascular bed caused by partial occlusion of the pulmonary arteries due to organized persistent thrombi, often accompanied by the remodeling of patent resistive pulmonary arterioles [8]. Based on registry data, the prevalence of CTEPH is approximately 25.8–38.4 per million [9,10,11]. Balloon pulmonary angioplasty (BPA) is a minimally invasive procedure that has become an effective treatment option for CTEPH patients who are ineligible for pulmonary endarterectomy (PEA) or present with persistent PH after surgery [8,12,13,14].

Sex-specific differences in CTEPH have been studied, with some evidence suggesting that women may be at higher risk for developing CTEPH [15]. Some studies suggest that there may be differences in the clinical presentation, risk factors, and outcomes of CTEPH treated by PEA between males and females [16]. However, there is limited research on the differences between women and men in CTEPH treated with BPA.

Therefore, the aim of this study was to evaluate sex-specific differences in patients with CTEPH who were treated with BPA procedures.

## 2. Materials and Methods

### 2.1. Study Design and Settings

This retrospective study included patients consulted by the CTEPH team in the Department of Pulmonary Circulation, Thromboembolic Diseases, and Cardiology between October 2011 and September 2020. Medical history and clinical data were obtained retrospectively from the patients’ medical records. As this was a retrospective study, patients were routinely diagnosed and treated, and no additional interventions were performed. A positive opinion from the Bioethics Committee was obtained (L.dz.OIL/KBL/27/2018).

Within routine patient management, the CTEPH team provided multi-specialist consultations, established the CTEPH diagnosis based on invasive measurement of hemodynamic parameters during right heart catheterization (RHC) [17] and results of imaging studies, and then referred patients to appropriate treatment methods: PEA, BPA, medical therapy, or combined therapy involving more than one of the mentioned methods according to current guidelines [8,18,19,20].

Data regarding the patients’ characteristics were evaluated including age, anthropometric data, comorbidities, anticoagulant and supporting treatment, and reasons for rejection from PEA. Hemodynamic data were obtained during RHC performed according to the current guidelines [17]. In addition, the functional class defined by the World Health Organization was noted, and results of the 6 min walking test (6-MWT) and Borg Dyspnea Scale ratings after 6-MWT were collected. Laboratory tests were performed including the levels of N-terminal pro-B-type natriuretic peptide (NT-proBNP)—reference value <125 pg/mL, high-sensitive troponin T—reference value <0.014 ng/mL, and creatinine—reference value <0.9 mg/dl for women and <1.20 mg/dl for men.

The patients’ data were analyzed at two points of time: at baseline and at follow-up. “Baseline” was defined as the moment of RHC performed before the first BPA procedure. “Follow-up” tests were performed three to six months after the last BPA procedure.

### 2.2. Statistical Analysis

Statistical analysis was performed with Statistica PL software (version 13, StatSoft, Tulsa, OK, USA). Categorical variables were presented as numbers and percentages. Continuous variables were presented as mean and standard deviation or median with interquartile range, depending on the distribution of the analyzed variable assessed using the Shapiro–Wilk test. The *t*-test was performed for data with a normal distribution, while the Mann–Whitney U-test was performed for data that did not follow a normal distribution. These tests were used to compare quantitative variables between groups. The Chi-square test for categorized variables was used to determine the differences between groups. For categorical variables with more than two categories, the Chi-square test was also used. Statistical significance in this study was established at *p* < 0.05.

## 3. Results

### 3.1. Patients

**Figure 1:**
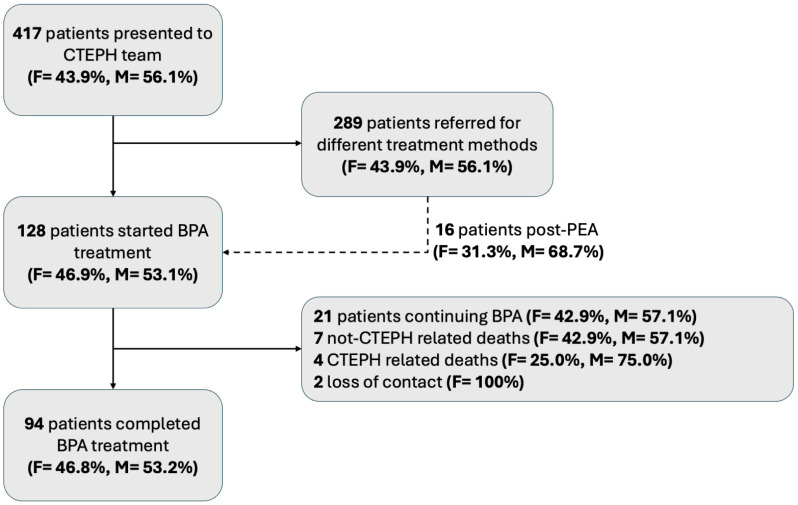
Flow chart of patient selection for this study. BPA—balloon pulmonary angioplasty; CTEPH—chronic thromboembolic pulmonary hypertension; F—female, M—male.

Of the 417 consulted patients, 128 were included in this study (Figure 1). In 21 patients, subsequent BPA sessions were planned; in 11 cases, due to the patient’s mortality, the BPA series was unfinished, and follow-up was not performed. Contact loss occurred in two cases before the treatment with BPA was completed. Hence, the final population included 94 patients who terminated BPA treatment and had follow-up tests performed after the last BPA session. Among them, there were 44 (46.8%) females and 50 (53.2%) males.

### 3.2. Evaluation at Baseline

Table 1 summarizes the baseline characteristics and clinical data stratified by gender. The women were nominally younger than the men (median age 54 (47–70) years vs. 66 (54–73) years), though the difference did not reach statistical significance (*p* = 0.07). The men were significantly taller, heavier, and had a greater body surface area, consistent with general population trends, while BMI was similar between the genders (*p* = 0.19).

Regarding comorbidities, chronic obstructive pulmonary disease (COPD) was observed exclusively in the men (26%), a finding that aligns with known gender differences in COPD prevalence and may contribute to differences in disease presentation and response to treatment. Other comorbidities showed no significant differences between the groups.

Anticoagulant treatment patterns were also similar, with the majority of both the women (54.6%) and men (54.0%) using DOACs, reflecting their widespread adoption in CTEPH management and alignment with current treatment guidelines. There were no significant differences in the use of specific pulmonary hypertension (PH) therapies, with sildenafil or riociguat being used by over 78% of both groups. The reasons for rejection from pulmonary endarterectomy (PEA) were comparable, with distal pulmonary vascular obstruction being the most common cause in both the women (63.6%) and men (52.0%).

Most of the women and men presented symptoms of III and IV WHO functional classes at baseline. There was no difference between the women and men regarding the severity of dyspnea assessed with the Borg Scale after 6-MWT. The men presented higher serum levels of creatinine (Table 2).

Hemodynamic measurements at baseline (Table 3) showed that the women had higher values of systolic pulmonary arterial pressure (sPAP) and pulmonary vascular resistance (PVR). In turn, the men had higher stroke volume (SV).

### 3.3. Evaluation at Follow-Up

After BPA procedures, there were no differences in the number of sessions (median: 5; IQR: 4–7), the number of treated vessels during one session (mean: 7, SD 2.5), or the required amount of contrast (mean: 253 mL, SD 42.8) and radiation (median: 146 mGy; IQR: 90–248) between the men and women. There were no differences in WHO functional classes of CTEPH regarding gender with most females and males presenting WHO class I or II after the BPA treatment was finished. Serum levels of troponin and creatinine were significantly higher in the men than in the women. Evaluation with the Dyspnea Borg Scale after 6-MWT revealed some differences between the sexes—94.9% of the women reported none or mild dyspnea while 20.4% of the men reported moderate to rather/very intense dyspnea (Table 4).

The post-treatment RHC data are presented in Table 5. The men had higher sPAP compared to the women, as well as pulmonary arterial wedge pressure. In turn, the women had a higher mean value of the cardiac index.

### 3.4. Treatment Outcomes

**Figure 2:**
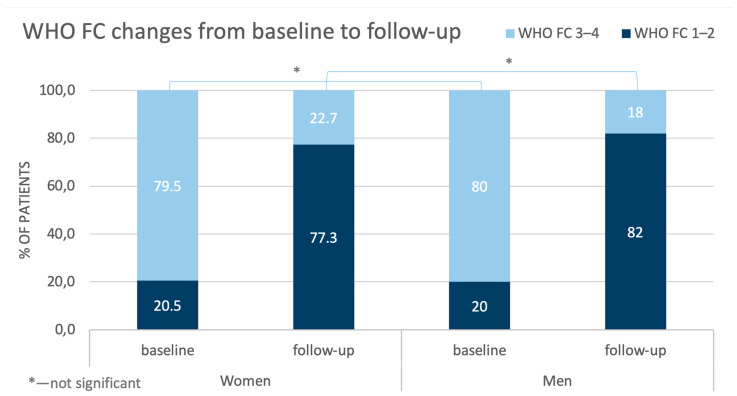
Comparison of changes in WHO FC in women and men from baseline to follow-up. WHO FC—World Health Organization functional class.

Analyzing the results of the CTEPH treatment with BPA, the classification of the patients according to WHO class improved. The proportions of both the women and men being classes I-II and III-IV were reversed at follow-up when compared with the baseline outcomes (Figure 2).

**Figure 3:**
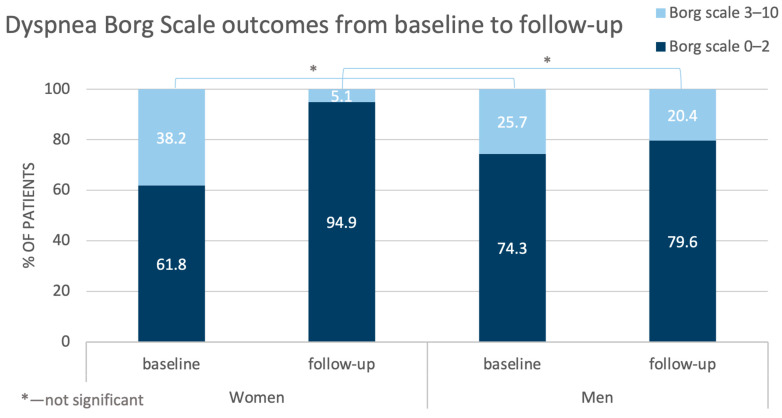
Comparison of changes in the outcomes of the Dyspnea Borg Scale in the women and men from baseline to follow-up.

Also, the outcomes of the Borg Dyspnea Scale improved, but the benefits were more pronounced for the women than for the men (Figure 3).

**Figure 4:**
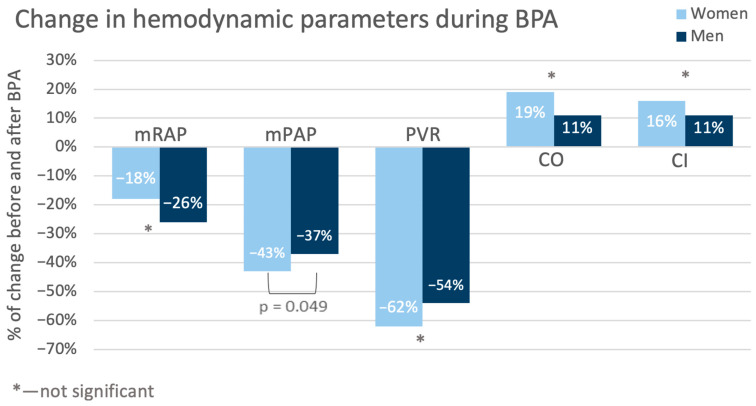
Changes in the values of hemodynamic variables during treatment (from baseline to follow-up). CI—cardiac index; CO—cardiac output; mPAP—mean pulmonary artery pressure; mRAP—mean right atrial pressure; PVR—pulmonary vascular resistance.

Additionally, a detailed analysis of RHC results at baseline and at follow-up was performed. The percentage changes in the hemodynamic variables are graphically presented in Figure 4.

The values (both nominal and percentage) of mean pulmonary arterial pressure (mPAP) in the women decreased more than in the men (−20.9 mmHg vs. −15.8 mmHg, *p* = 0.04; −43% vs. −37%, *p* = 0.049). Also, pulmonary vascular resistance (PVR) in the women was nominally lower than in the men. But this observation pertained only to the nominal values (−6.64 j.W. vs. −3.85 j.W. *p* = 0.048; −62% vs. −54%, *p* = 0.12). Detailed hemodynamic variable changes are presented in Table 5.

## 4. Discussion

CTEPH is a rare condition, but it can lead to right heart failure, multiorgan disfunction, and death if left untreated. The BPA therapy that has been used relatively recently in the treatment of patients with CTEPH [21,22] has been upgraded in the recent guidelines for CTEPH treatment, which currently recommend BPA as a part of a multimodal approach for patients who have inoperable lesions or have residual PH after PEA and distal obstructions amenable to BPA (class of recommendation IB). This procedure may also be applied to those patients who are operable but have a high proportion of distal disease, where a PEA procedure may generate a high risk for them. BPA can also be considered in some symptomatic patients with CTEPD without PH [8].

The differences observed in patients with CTEPH treated with BPA may vary significantly across populations due to genetic, environmental, and healthcare access factors. For example, in Japan, a notably higher proportion of female patients (75%) undergoing BPA has been reported, in contrast to the more balanced gender distributions observed in other regions [23]. Similarly, a registry study covering countries such as Russia, Kazakhstan, Turkey, Lebanon, and Saudi Arabia found that most patients presented with advanced disease at baseline, indicating potential delays in diagnosis and differences in disease progression compared to European cohorts [24]. These findings underscore the influence of regional variations, including genetic predispositions, environmental factors, and healthcare practices, on patient demographics and treatment outcomes. Consequently, while our study provides valuable insights into a European cohort, caution is warranted when extrapolating these findings to non-European populations, as local factors may substantially impact disease presentation and management outcomes.

A recent study that analyzed the preoperative computed tomography pulmonary angiography of patients who underwent PEA for CTEPH identified sex-specific differences in surgical cases [25]. Men had more vessels involved than women (mean 20.3 vs. 17.1, *p* = 0.004) and had fewer disease-free pulmonary segments (mean 4.9, SD 4.3 vs. 7.6, SD 5.5, *p* = 0.001). In addition, men had a greater number of webs, eccentric thickening, and occlusions. The distribution of lesion type did not significantly differ between the sexes at the main or lobar level, but men had significantly more lesions in the segmental vasculature while women had a higher proportion of subsegmental lesions (*p* < 0.001) despite no significant differences in baseline hemodynamics [25]. Although it was described that after PEA, women benefit less from the reduction in PVR (437 Dynes∙s∙cm−5 vs. 324 Dynes∙s∙cm−5 in males, *p* < 0.01), the overall 10-year survival after surgical treatment was similar (73% in females vs. 84% in males, *p* = 0.08) [26]. In the multivariate analysis, the female sex remained as an independent factor affecting the need for targeted PH medical therapy after PEA (HR 2.03, 95%CI 1.03–3.98, *p* = 0.04), which suggests other mechanisms may be responsible for the worse response to surgical treatment in females with proximal disease [26]. In addition, another study analyzed gender-dependent differences in CTEPH patients treated with PEA from a European registry [16]. The study showed that women had better survival rates than men regardless of whether they underwent PEA or not. However, data on other treatment modalities for CTEPH are also limited.

**Figure 5:**
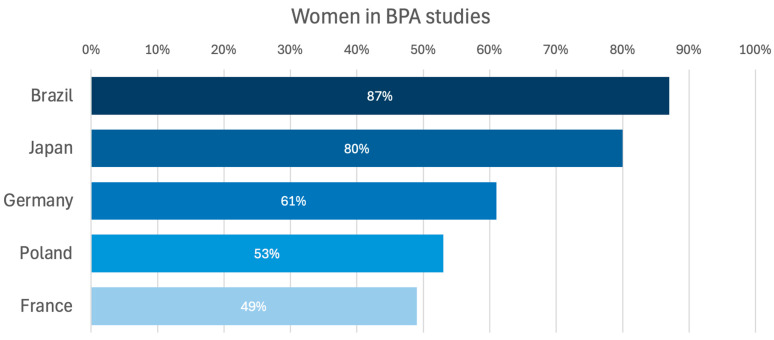
Percentage of women represented in BPA studies [14,19,30,31,32]. BPA—balloon pulmonary angioplasty.

There is a lack of data on whether sex affects BPA results. Therefore, we aimed to evaluate whether differences exist between men and women with CTEPH and to evaluate if the outcomes of treatment with BPA differ regarding sex/gender. Women are known to be more susceptible to PH than men and, therefore, usually among patients with CTEPH, but on the other hand, women also have better survival [16,27,28]. In the case of CTEPH, the latest publication based on the European registry indicated an equal ratio of affected women and men [16], similar to the US registry [29]. The registry developed by the International CTEPH Association reported a general dominance of women (52.4%); however, in Europe, they made up slightly less than half (48.7%) of the patients with CTEPH [23]. Similar trends are corroborated by data from BPA registries, which consistently highlight variations in gender representation across different cohorts. The highest proportion of female participants was observed in the Brazilian registry, where women accounted for 87% [30] of the cohort. Similarly, in the Japanese registry, women constituted 80% of the participants [19], demonstrating a significant female predominance in this population. In contrast, in European BPA registries, the proportion of women ranged from 49% to 61% [14,31,32] (Figure 5). These differences may reflect regional variations in disease prevalence or referral patterns.

The European registry indicated that women had a lower prevalence of some CV risk factors than men, such as previous acute coronary syndrome, smoking, and COPD, but they more often suffered from obesity and had cancer or thyroid disease history [16]. The women and men included in our study did not differ in terms of comorbidities, except for the occurrence of COPD, which was much less frequently diagnosed in the women (26% vs. 0%). This disparity could introduce a bias in the assessment of dyspnea severity and other parameters both pre- and post-BPA. COPD is known to contribute to Group 3 PH, which may further complicate the interpretation of the observed differences between the sexes. While these findings highlight the complexity of pulmonary hemodynamics in patients with concomitant conditions, they also underscore the need for careful consideration of such confounding factors in future research.

As CTEPH is strongly associated with the occurrence of pulmonary embolism and an incomplete thrombus resolution [33], sex/gender differences related to coagulation should be also considered. Typical thrombogenic factors were not proven to increase in patients with CTEPH in contrast to plasma factor VIII [34,35]. This factor physiologically has higher values in women than in men [36], which may predispose women to developing CTEPH. In the Japanese BPA Registry, females represented 80% of the included patients, and previous episodes of acute pulmonary embolism and deep vein thrombosis were not frequent (15.3% and 43.7%, respectively) [19], as compared with reports from other Western countries (74.8% and 58.1%, respectively, in a European registry) [37].

Assessing the patients’ status at baseline, we found that the women tended to have worse hemodynamics values than the men, as indicated by higher sPAP and PVR and by lower SV. Creatinine levels were slightly higher in the men, but this probably resulted from the physiologically higher muscle mass in the men. In turn, clinical presentation in the women and men was quite similar—there were no significant differences between sex/gender in the results of the Dyspnea Borg Scale or in the distance walked during 6-MWT. Our population also did not differ in terms of WHO FC, and most patients were diagnosed with WHO FC class 3. This observation is consistent with the results described in other studies [23,28,38] and also with observations from the European CTEPH registry [16], indicating that women slightly more often have functional capacity class III/IV diagnosed than men. More severe courses of the disease in women than men with CTEPH were also reported by Wu et al. [39].

More severe baseline hemodynamic parameters in women compared with men together with similar clinical symptomatology suggest that at diagnosis, women are better adapted to the disease than men. On the other hand, it may be hypothesized that women will require more BPA sessions to achieve similar improvements in hemodynamics as men.

There are no clearly defined therapeutic targets for BPA treatment in patients with CTEPH. The guidelines suggest (i) achieving a good functional class (WHO-FC I–II), (ii) improving hemodynamic parameters, and (iii) enhancing quality of life [8]. However, some recent data from the ESC Working Group Statement defined a hemodynamic BPA treatment goal to achieve final mPAP < 30 mmHg [12]. Comparing the effects of BPA from two multicenter registries (Japanese and Polish) and from single-expert centers (German and French), it was demonstrated that only Japanese patients were able to reach the defined BPA treatment goal of mPAP below 30 mmHg [40]. This may be due to the intrinsic differences between European and Japanese patients, with European CTEPH patients having higher serum concentrations of C-reactive protein, fibrinogen, and myeloperoxidase and more red thrombus than Japanese CTEPH patients [41]. However, the high-volume representation of women in the Japanese registry may be also suggestive of gender-related outcomes in BPA treatment.

The control tests performed in the studied population after completion of BPA treatment revealed better hemodynamic status (especially regarding mSAP, sPAP, PCWP, and CI) in the women than in the men, although at baseline, the situation was the opposite. The detailed analysis demonstrated that after treatment, many parameters changed more in the women than in the men, with decreases in mPAP and PVR being the most pronounced. These were reflected particularly in the outcomes of the Dyspnea Borg Scale—compared with baseline at follow-up, in about 30% of the women, the ratings shifted towards points 0–2, and such an improvement was observed only in about 5% of the men. This difference did not achieve statistical significance in the results of the 6-MWT.

Our results are even more interesting considering that there were no differences in the number of sessions, the number of treated vessels, or the required amount of contrast and radiation between the men and women. This may suggest that women respond better to CTEPH treatment with BPA than men.

The above observation seems to be in line with the previously reported better long-term survival in women compared with men. This phenomenon is suggested to be related to better right ventricular function in females than in males [42,43]. Unfortunately, echocardiographic data were unavailable in our study; hence, we were unable to assess right ventricular functions in our population. However, hemodynamic differences exist between different types of CTEPH with a worse condition in the central than the peripheral form of the disease [44]. In addition, in some studies, women tended to deteriorate more than males during follow-up [45].

Based on our results, we believe that the treatment strategy for CTEPH should be more tailored to specific patient characteristics, including sex. While the women in our cohort seem to exhibit more favorable outcomes with BPA, such as better hemodynamic responses, it is important to acknowledge that men may require a more individualized approach to achieve similar results. This could involve optimizing the BPA technique with more intensive and frequent treatment sessions to improve their outcomes. However, it is crucial to note that the current evidence is preliminary, and more data are needed to determine whether sex-specific treatment guidelines should be prioritized. Additionally, while some studies suggest better long-term survival in women, further investigation into survival time, symptom-free periods, and mortality is required to draw definitive conclusions on the need for earlier intervention for women. Consequently, clinical decision-making should be guided by a comprehensive assessment of each patient’s unique characteristics, and not solely on sex differences at this stage.

## Strengths and Limitations of This Study

To our knowledge, this is the first very detailed study analyzing the impact of patients’ sex/gender on the results of BPA therapy in patients with CTEPH. As it was a retrospective (single center, small group) study, we were able to provide valuable data from real clinical practice. However, some assessments are missing, which is an inherent limitation to this type of research. The retrospective design of this study made it possible to rely exclusively on existing data, and the intervention was not blinded at any stage.

## 5. Conclusions

Although many studies demonstrated that BPA improves both the hemodynamic parameters and clinical status of patients with CTEPH [46,47], sex/gender-specific treatment results are unknown. Also, the current guidelines do not promote any sex/gender-specific approach, which implies that the diagnostic–therapeutic process is the same in women and men. Therefore, our data may be used to initiate further studies analyzing different scenarios of clinical management depending on sex/gender. We observed that at diagnosis, women had more severe hemodynamic parameters than men, which were accompanied by similar clinical symptomatology in both sexes. Despite this, women’s hemodynamic status was better after BPA therapy. The above outcomes may suggest that at diagnosis, women are better adapted to the disease than men, and women also respond better to BPA treatment. However, we are aware that these data are just preliminary, and research evidence from randomized controlled trials is needed to demonstrate whether the management of a patient with CTEPH should be sex-differentiated.

## Figures and Tables

**Table 1:** Characteristics of patients at baseline.

Variable		Female (*n* = 44)	Male (*n* = 50)	*p*-Value
**Age, years**		54 (47–70)	66 (54–73)	0.07
**Anthropometric measures**	Weight, kg	67 (61–74)	81 (74–93)	<0.001
	Height, cm	162 (7.8)	175 (7.4)	<0.001
	BSA, m^2^	1.73 (0.18)	1.98 (0.17)	<0.001
	BMI, kg/m^2^	25.6 (23.3–28.4)	26.8 (24.5–29.9)	0.19
**Comorbidities**	Previous venous thrombosis, %	19 (43.2)	26 (52.0)	0.42
	Previous acute pulmonary embolism, %	37 (84.1)	36 (72.0)	0.22
	Hypertension, %	19 (43.2)	28 (56.0)	0.30
	Diabetes, %	2 (4.6)	7 (14.0)	0.23
	Coronary artery disease, %	9 (20.5)	14 (28.0)	0.47
	Hyperlipidemia, %	13 (29.6)	19 (38.0)	0.51
	Chronic obstructive pulmonary disease, %	0 (0.0)	13 (26.0)	<0.001
	Chronic kidney disease, %	8 (18.2)	10 (20.0)	1.00
	Atrial fibrillation, %	4 (9.1)	12 (24.0)	0.10
	Inferior vena cava filter, %	6 (13.6)	5 (10.0)	0.75
**Anticoagulant treatment**	VKA, %	12 (27.3)	14 (28.0)	0.75
	LMWH, %	7 (15.9)	9 (18.0)	
	DOAC, %	24 (54.6)	27 (54.0)	
**PH specific therapy**	Sildenafil or riociguat, %	36 (81.8)	39 (78.0)	0.71
	Oxygen therapy, %	8 (18.2)	7 (14.0)	0.58
**Reasons for rejection from PEA**	Distal pulmonary vascular obstruction, %	28 (63.6)	26 (52.0)	0.33
	Comorbidities, %	6 (13.6)	10 (20.0)	
	Lack of patient consent, %	5 (11.4)	3 (6.0)	
	Post-PEA CTEPH, %	5 (11.4)	11 (22.0)	

Data are presented as *n* (%), median (interquartile range), or mean (SD). BMI—body mass index; BSA—body surface area; DOAC—direct oral anticoagulants; PEA—pulmonary endarterectomy; PH—pulmonary hypertension; LMWH—low-molecular-weight heparin; SD—standard deviation; VKA—vitamin K antagonists.

**Table 2:** Patients’ clinical status at baseline.

Variable		Female (*n* = 44)	Male (*n* = 50)	*p*-Value
Heart rate, bpm		77 (66–92)	72 (65–78)	0.09
WHO FC, *n* (%)	1	0	0	0.69
	2	9 (20.5)	10 (20.0)	
	3	32 (72.7)	34 (68.0)	
	4	3 (6.8)	6 (12.0)	
6-MWT, m		341 (135.8)	366 (135.3)	0.43
Borg Dyspnea Scale, points (%)	0	17 (50.0)	22 (62.9)	0.89
	1	0	0	
	2	4 (11.8)	4 (11.4)	
	3	5 (14.7)	3 (8.6)	
	4	2 (5.9)	1 (2.9)	
	5	3 (8.8)	3 (8.6)	
	6	0	0	
	7	0	0	
	8	3 (8.8)	2 (5.7)	
	9	0	0	
	10	0	0	
Laboratory tests	NT-proBNP, pg/mL	1195 (412–1970)	1367 (652–2533)	0.71
	Troponin T, ng/mL	0.011 (0.006–0.021)	0.015 (0.009–0.026)	0.07
	Creatinine, mg/dL	0.86 (0.80–0.98)	1.13 (0.99–1.30)	<0.001

Data are presented as *n* (%), median (interquartile range), or mean (SD). NT-proBNP—N-terminal pro-B-type natriuretic peptide; SD—standard deviation; WHO FC—World Health Organization functional class; 6-MWT—6 min walking test.

**Table 3:** Patients’ hemodynamic status at baseline.

Variable	Female (*n* = 44)	Male (*n* = 50)	*p*-Value
mRAP, mmHg	7 (5–10)	9 (5–12)	0.31
sPAP, mmHg	85 (17.6)	76 (18.5)	0.03
dPAP, mmHg	29 (7.9)	28 (8.1)	0.56
mPAP, mmHg	49 (10.7)	45 (10.5)	0.08
PCWP, mmHg	9 (6–12)	10 (9–12)	0.30
CI, L/min/m^2^	2.24 (1.81–2.80)	2.24 (1.92–2.50)	0.70
SV, mL/beat	54 (17.2)	64 (17.5)	0.01
SVI, mL/beat/m^2^	31.59 (9.9)	31.91 (7.9)	0.86
PVR, Wood Units	9.89 (6.31–14.06)	8.21 (5.55–10.17)	0.03

Data are presented as median (interquartile range) or mean (SD). CI—cardiac index; dPAP—diastolic pulmonary arterial pressure; mPAP—mean pulmonary artery pressure; mRAP—mean right atrial pressure; PCWP—pulmonary capillary wedge pressure; PVR—pulmonary vascular resistance; SD—standard deviation; sPAP—systolic pulmonary arterial pressure; SV—stroke volume; SVI—stroke volume index.

**Table 4:** Patients’ clinical outcomes at follow-up.

Variable		Female (*n* = 44)	Male (*n* = 50)	*p*-Value
Heart rate, bpm		68 (61–78)	67 (59–78)	0.53
WHO FC, *n* (%)	1	14 (31.8)	13 (26.0)	0.59
	2	20 (45.5)	28 (56.0)	
	3	10 (22.7)	9 (18.0)	
	4	0	0	
6-MWT, m		428 (134)	444 (134)	0.57
6-MWT, Δm		+93 (96.6)	+76 (108)	0.47
Borg Dyspnea Scale, points (%)	0	34 (87.2)	35 (79.6)	0.03
	1	0	0	
	2	3 (7.7)	0	
	3	0	2 (4.5)	
	4	0	1 (2.6)	
	5	0	4 (10.5)	
	6	0	1 (2.6)	
	7	2 (5.1)	0	
	8	0	0	
	9	0	0	
	10	0	0	
Laboratory tests	NT-proBNP, pg/mL	152 (87–376)	223 (73–743)	0.60
	Troponin T, ng/L	0.007 (0.004–0.012)	0.014 (0.009–0.022)	<0.001
	Creatinine, mg/dL	0.79 (0.72–0.90)	1.05 (0.96–1.20)	<0.001

Data are presented as *n* (%), median (interquartile range), and mean (SD). NT-proBNP—N-terminal pro-B-type natriuretic peptide; WHO FC—World Health Organization functional class; 6-MWT—6 min walking test.

**Table 5:** Patients’ hemodynamic outcomes at follow-up and changes in some variables from baseline to follow-up.

Variable		Female (*n* = 44)	Male (*n* = 50)	*p*-Value
mRAP, mmHg		5 (4–7)	6 (3–7)	0.77
sPAP, mmHg		43 (37–49)	49 (41–54)	0.04
dPAP, mmHg		15 (12–18)	17 (13–22)	0.05
mPAP, mmHg		26 (22–30)	29 (23–33)	0.11
	Δnominal	−20.9 (±12.2)	−15.8 (±10.8)	0.04
	Δ%	−43% (−57–25)	−37% (−47–−18)	0.049
PCWP, mmHg		9 (8–11)	10 (9–14)	0.03
CI, L/min/m^2^		2.82 (0.50)	2.57 (0.53)	0.03
	Δnominal	+0.40 (±0.77)	+0.30 (±0.50)	0.42
	Δ%	+16% (−1–51)	+11% (−5–32)	0.34
SV, mL/beat		70.87 (14.37)	76.62 (20.02)	0.12
SVI, mL/beat/m^2^		40.77 (7.61)	38.33 (9.21)	0.17
PVR, Wood Units		3.34 (2.63–3.87)	3.08 (2.58–4.49)	0.65
	Δnominal	−6.64 (−10.28–−2.85)	−3.85 (−6.48–−1.77)	0.048
	Δ%	−62% (−79–−42)	−54% (−69–−36)	0.12

Data are presented as median (interquartile range) or mean (SD). CI—cardiac index; dPAP—diastolic pulmonary arterial pressure; mPAP—mean pulmonary artery pressure; mRAP—mean right atrial pressure; PCWP—pulmonary capillary wedge pressure; PVR—pulmonary vascular resistance; SD—standard deviation; sPAP—systolic pulmonary arterial pressure; SV—stroke volume; SVI—stroke volume index.

## Data Availability

The original contributions presented in this study are included in the article. Further inquiries can be directed to the corresponding author.

## Abbreviations

The following abbreviations are used in this manuscript:

BPA	balloon pulmonary angioplasty
CTEPH	chronic thrombo-embolic pulmonary hypertension
CVD	cardiovascular diseases
RHC	right heart catherization
PEA	pulmonary endarterectomy
6-MWT	6 min walking test
NT-proBNP	N-terminal pro-B-type natriuretic peptide
M	male
F	female
BMI	body mass index
DOAC	direct oral anticoagulants
PH	pulmonary hypertension
SD	standard deviation
VKA	vitamin K antagonists
LMWH	low-molecular-weight heparin
WHO FC	World Health Organization functional class
mRAP	mean right atrial pressure
sPAP	systolic pulmonary arterial pressure
dPAP	diastolic pulmonary arterial pressure
mPAP	mean pulmonary artery pressure
PCWP	pulmonary capillary wedge pressure
CI	cardiac index
SV	stroke volume
SVI	stroke volume index
PVR	pulmonary vascular resistance
IQR	interquartile range
COPD	chronic obstructive pulmonary disease
